# Toward developing a metastatic breast cancer treatment strategy that incorporates history of response to previous treatments

**DOI:** 10.1186/s12885-021-07912-7

**Published:** 2021-03-01

**Authors:** Aleksandra K. Olow, Laura van ’t Veer, Denise M. Wolf

**Affiliations:** 1 Department of Laboratory Medicine, Helen Diller Family Comprehensive Cancer Center, University of California San Francisco, San Francisco, CA 94115 USA; 2 Merck Research Laboratories, 213 E Grand Avenue, South San Francisco, CA 94080 USA

**Keywords:** Metastatic breast cancer, Resistance, Recommendation algorithm

## Abstract

**Background:**

Information regarding response to past treatments may provide clues concerning the classes of drugs most or least likely to work for a particular metastatic or neoadjuvant early stage breast cancer patient. However, currently there is no systematized knowledge base that would support clinical treatment decision-making that takes response history into account.

**Methods:**

To model history-dependent response data we leveraged a published in vitro breast cancer viability dataset (84 cell lines, 90 therapeutic compounds) to calculate the odds ratios (log (OR)) of responding to each drug given knowledge of (intrinsic/prior) response to all other agents. This OR matrix assumes (1) response is based on intrinsic rather than acquired characteristics, and (2) intrinsic sensitivity remains unchanged at the time of the next decision point. Fisher’s exact test is used to identify predictive pairs and groups of agents (BH *p* < 0.05). Recommendation systems are used to make further drug recommendations based on past ‘history’ of response.

**Results:**

Of the 90 compounds, 57 have sensitivity profiles significantly associated with those of at least one other agent, mostly targeted drugs. Nearly all associations are positive, with (intrinsic/prior) sensitivity to one agent predicting sensitivity to others in the same or a related class (OR > 1). In vitro *conditional* response patterns clustered compounds into five predictive classes: (1) DNA damaging agents, (2) Aurora A kinase and cell cycle checkpoint inhibitors; (3) microtubule poisons; (4) HER2/EGFR inhibitors; and (5) PIK3C catalytic subunit inhibitors. The apriori algorithm implementation made further predictions including a directional association between resistance to HER2 inhibition and sensitivity to proteasome inhibitors.

**Conclusions:**

Investigating drug sensitivity conditioned on observed sensitivity or resistance to prior drugs may be pivotal in informing clinicians deciding on the next line of breast cancer treatments for patients who have progressed on their current treatment. This study supports a strategy of treating patients with different agents in the same class where an associated sensitivity was observed, likely after one or more intervening treatments.

**Supplementary Information:**

The online version contains supplementary material available at 10.1186/s12885-021-07912-7.

## Background

**Fig. 1:**
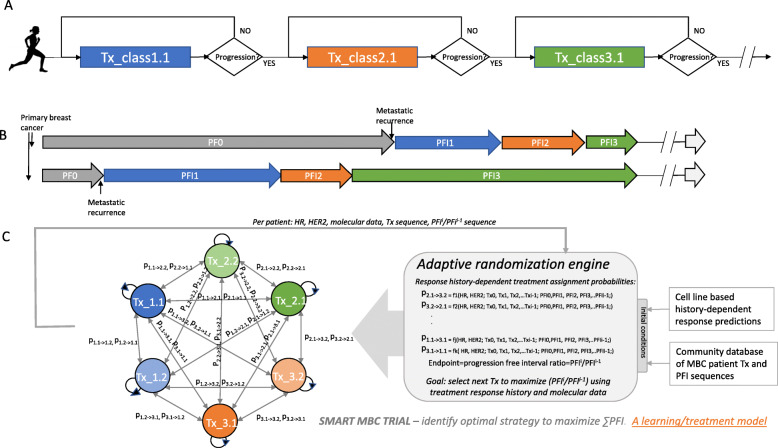
Transitioning to a MBC treatment model incorporating history of response. **a** Current treatment model for MBC, where patients switch treatments upon progression following NCCN guidelines and physician choice. **b** Two example patient trajectories, the top showing a typical sequence with progressively decreasing progression free intervals (PFI) from the time of metastatic recurrence. The bottom illustrates a potential departure, with a longer PFI (green) following a shorter PFI (orange). In the top trajectory, the progression free interval PFI^i^/PFI^i-1^ is always < 1, whereas in the bottom, PFI^i^/PFI^i-1^ > 1 for a transition. **c** Schematic showing a learning/treatment model for MBC. The left-hand side shows a network of possible treatments, where each therapeutic class has multiple agents (e.g., Tx_1.2 = treatment Class 1, agent 2). Upon progression, patients being treated with Agent I in Class J (Tx_i.j) can potentially transition to any other treatment in the network. Transitions are selected based on probabilities generated by an adaptive randomization engine (right). This engine generates predictive probabilities based on the patient’s treatment-response history and molecular phenotype using (1) cell line-based phenotype- and history-dependent response predictions, and (2) a community database of MBC patient Tx and PFI sequences. All treatment-PFI sequences are fed back into the engine to refine predictions. Within the engine, the endpoint is progression free interval ratio PFI^i^/PFI^i-1^. At a higher level of resolution/abstraction, this schema constitutes a SMART MBC learning/treatment system with the goal of identifying the optimal strategy to maximize PFI^i^/PFI^i-1^ (short term) and ∑PFI (long term) using response history and molecular tumor features. In a trial setting the comparator would either be the typical treatment trajectory for MBC patients (A), or possibly the same treatment network with equal probabilities assigned to all treatment transitions, adjusting for subtype

Once breast cancer has spread to distant sites, it is considered to be an incurable disease leading to death. The development of new drugs for managing breast cancer has led to longer life-spans for women with some types of Stage IV disease, such ER+ and Her2+ subtypes, but less so for others, including those with BRCA1/2 wildtype triple negative (TN) disease [1, 2]. What nearly all women with Stage IV disease have in common is that once diagnosed with metastatic recurrence, they remain in treatment for life. A typical treatment course for women with ER+ MBC is to first cycle through all the endocrine treatments, and then transition to cytotoxic chemotherapies or endocrine/targeted therapies when endocrine treatments are no longer effective (Fig. 1a) [3]. Women with TN disease are on cytotoxic chemotherapies from the time of first recurrence, moving from drug to drug and protocol to protocol upon progression. For all, managing patient care is a matter of selecting drugs or drug combinations to be given in sequence, ideally using the response history of the individual patient and associations mined over the population in addition to molecular features to select the next treatment after the current one fails, with the object of prolonging life while maintaining quality of life (Fig. 1b).

Unfortunately, such a rational, history-dependent, data-supported algorithm for treatment selection does not currently exist. Though there have been many clinical trials comparing outcome and response rates for particular drugs [4–6], these trials tend to be narrowly focused and short term. They do not address long-term strategies or practical questions faced by every medical oncologist treating metastatic breast cancer patients. For example, if a patient initially responded well to a drug in class A, but progressed quickly on a drug in class B, what is the best-odds choice for the next treatment, and/or how to prioritize among clinical trial options?

Currently, there is a lack of high-throughput sequential and combinatorial treatment data supporting rigorous investigation of this question over a wide range of therapeutics. However, high throughput drug sensitivity data on cell line panels permits investigation of the more constrained question: ‘if the past history of response of a tumor to a particular agent or set of agents is known, and if the tumor has not been sufficiently altered by subsequent treatments to change its likelihood of responding to a new agent, what is the likelihood of sensitivity to this agent?’. In other words, what does a cancer’s treatment response history tell us about its *intrinsic* sensitivity or resistance to compounds it has not yet been treated with, and how can we use this information to make a ‘history-informed guess’ as to the best treatment to try next?

As a first step in this direction, we utilized public in vitro viability panels with 90 experimental or approved therapeutic compounds and 84 breast cancer cell lines representing 35 luminal, 27 basal, 10 claudin-low, 9 normal and 3 of unknown subtypes, with 27 of them ERBB2 amplified [7]. At the time of this publication, 39 of the studied compounds have been approved by the US Food and Drug Administration, including eight for breast cancer, as indicated by the NIH, National Cancer Institute website. To uncover patterns of drug response, we used both standard response pattern clustering and statistical association methods and applied data mining algorithms like those used in online advertising technology by companies like Amazon or Google, to recommend products customers are likely to want based on their purchasing histories. Association mining with recommender systems is an efficient algorithm which is not only capable of handling big datasets, but importantly provides both an exhaustive, and easy to interpret list of rules. With the growing amount of high-throughput drug screening panels, this approach offers a scalable solution to model complex multi-way relationships between drugs. Prior research has showcased the use of similar approaches space to predict drug responses for specific unseen cell-lines and patient based on their intrinsic transcriptional or molecular profiles [8–11]. We show that statistical association analysis and recommendation systems can be used to support rational treatment decision-making that takes into account the individual patient drug response history alone. This approach can be easily extended to serial exposure data capturing acquired sensitivity or resistance once such high throughput datasets become available (Fig. 1c), and ideally would also incorporate a tumor’s molecular feature trajectory and is applicable to neoadjuvant chemo−/targeted-therapy trials for early-stage breast cancer seeking to use information on response to one therapy to determine the next with the eventual goal of pathologic complete response (pCR).

## Methods

### Dataset processing and annotation

Cell line viability published data from 84 breast cancer cell lines tested in triplicate against nine concentrations of 90 therapeutic compounds, including conventional cytotoxic agents such as well as targeted agents such as hormone and kinase inhibitors, often with overlapping activity [7] was used (Supplemental Table 1A). The cell line compendium is composed of 35 luminal, 27 basal, 10 claudin-low, 7 normal-like, 2 matched normal cell lines, and 3 of unknown subtype [7]. Fourteen luminal and seven basal cell lines were also ERBB2-amplified [7] (Supplemental Table 1A).

The concentration required to inhibit growth by 50% (GI50) was used as the response measure for each compound. To obtain a binarized response dataset, these drug responses were simplified as follows: the previously established GI50 dichotomization threshold for each compound [7] was used as a cut-off for treatment response encoded as a binary variable (sensitive = 1 or resistant = 0). For each drug, a generalized therapeutic target is manually annotated (Supplemental Table 1B) to categorize it by its primary mode of action.

### Odds ratio (log (OR)) matrix as a model for history-dependent response

In the absence of data derived from cells treated sequentially with multiple treatments, we model history-dependent response data as a matrix of odds ratios (log (OR)) of responding to one drug given knowledge of (intrinsic/prior) response to another drug, for all drugs in the panel. We refer to this association between two compounds as conditional sensitivity. Prior to constructing the OR matrix, cell lines with more than 50 missing GI50 values were excluded from the binary response dataset yielding binarized response data for 90 drugs and 50 cell lines. For each agent, we used Fisher’s exact test to assess association between the response profile of the agent vs. all other agents, and applied Benjamini Hochberg multiple testing correction to *p*-values to assess significance (BH *p* < 0.05). This model assumes (1) past response to a drug, whether sensitive or resistant, is based on *intrinsic rather than acquired* characteristics and (2) a cell line (or patient’s) intrinsic sensitivity/resistance profile remains unchanged despite the passage of time and treatment, at the time of the next decision point. Parallel uncorrected analysis using only Fisher *p* < 0.05 rather than FDR-corrected BH p < 0.05 is also provided in Supplemental Figure S2.

### Clustering OR analysis

To explore the relationships between cell lines in terms of drug responses, as well as similarities between the examined compounds in the binarized drug response dataset, we clustered the binary data using binary distance matrix and a ward-clustering algorithm, and the continuous (log (OR)) data using correlation distance and complete-linkage clustering. The uncertainty in the hierarchical cluster analysis was calculated using Pvclust (v. 2.2.0), an add-on package for a statistical software R (v. 3.6.1) [12, 13]. We calculated probability values (*p*-values) for each cluster using multiscale bootstrap resampling techniques. Two types of p-values are calculated: approximately unbiased (AU) p-value and bootstrap probability (BP) value. The cutoff p-value for cluster selection is set at AU alpha = 0.9.

### Recommender systems

In order to predict the level of response for a cell line in a subsequent treatment, as well as create a list of the top recommended compound, we utilized a data mining method for making drug recommendations through a collaborative filtering algorithm. Shortly, this approach examines each cell line that responded to a particular drug and creates lists of other drugs they also responded to, calculating the confidence levels for each subsequent drug. Compounds that appear most frequently and meet confidence, lift and support criteria given a prior compound, become then a part of the drug recommendation rules. This approach creates a graphical network of four types of drug relationships: (1) if sensitive to drug A, then sensitive to drug B, (2) if sensitive to drug A, then resistant to drug B, (3) if resistant to drug A, then also resistant to drug B, and (4) if resistant to drug A then sensitive to drug B.

R language implementation of apriori recommendation system algorithm (arules package v 1.6.6) was used on a binary dataset of response of 70 cell lines to 90 therapeutic compounds, to obtain non-redundant recommendations i.e., rules with minimum confidence level of 0.8 and minimum support of 0.2. These cutoff points ensured that drugs with insufficient amount of information (support) are excluded, as well as that the established rules have a high probability to be correct for new recommendations (confidence). Only one-to-one drug relationships were considered for this analysis. For each mined rule we calculated values, which can be interpreted as the deviation of the support of the whole rule from the support expected under independence given the supports of the antecedent drugs/items (LHS) and the consequent drug recommendations (RHS) [14]. Higher lift values indicate stronger associations. The compound listed on the LHS of a rule, is the specified sensitivity/resistance constraint or condition for the sensitivity/resistance of the compound listed in the RHS of the rule. In other words, given the sensitivity/resistance to a LHS drug (“if”), we infer sensitivity/resistance to RHS drug (“then”).

## Results

### Clustering analysis of binarized drug response data reveals anti-cancer agents with similar patterns of response across breast cancer cell lines

The drug response matrix used in this study derives from published cell line viability data from 84 breast cancer cell lines tested in triplicate against nine concentrations of 90 therapeutic compounds, including conventional cytotoxic agents as well as targeted agents such as hormone and kinase inhibitors, often with overlapping activity [7]. The concentration required to inhibit growth by 50% (GI50) was used as the response measure for each compound.

As a first step in exploring the similarities and differences between cancer therapeutics, in terms of their patterns of sensitivity/resistance observed across breast cancer cell lines, we created a binarized response dataset using previously established GI50 dichotomization thresholds for each compound [7] and encoded treatment response as a binary variable (sensitive = 1 or resistant = 0). (See Methods for additional details.)

**Fig. 2:**
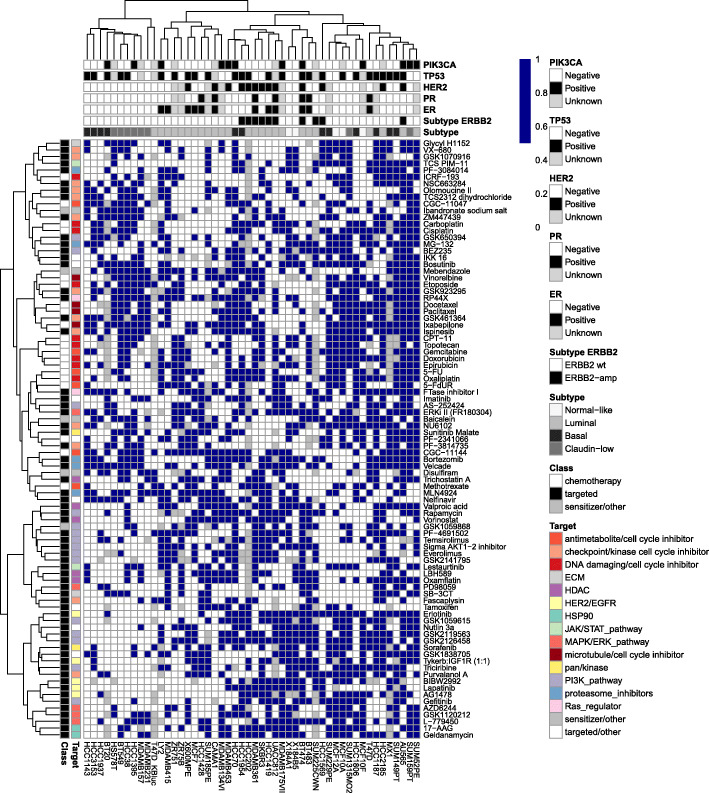
Relationships between transcriptional subtypes and response to specific drug classes represented in a heatmap of binary response values determined using a GI50 threshold (white color encoding resistance, dark blue – sensitivity); binary distance, ward clustering. Compounds with similar mechanisms of action cluster closely together. Luminal cell lines cluster separately from those of another transcriptional subtype. 50 cell lines with more than 50 numeric measurements are represented

Unsupervised hierarchical clustering of this binary drug response data matrix (Fig. 2, Supplemental Figure S1), examined along the columns, illustrates that cell lines show similar patterns of response based on related transcriptional subtype (e.g. most of luminal and ERBB2 amplified luminal cell lines cluster together). Claudin-low and certain basal subtypes appear most responsive to cell cycle and proteasome inhibitors, whereas luminal subtypes tend to be more sensitive to HDAC and PI3K pathway inhibitors.

Examined along the rows the heatmap in Fig. 2 reveals as expected, that many compounds with similar and related modes of action tend to cluster together. For instance, six out of seven PI3K pathway inhibitors cluster strongly together, and closely to a cluster containing all five of the HDAC inhibitors. As well, platinum drugs cisplatin, oxaliplatin, and carboplatin cluster together, as do microtubule/spindle inhibitors paclitaxel, ispinesib, and ixebepilone. Thus, breast cancer cells with intrinsic sensitivity to a drug in the PI3K, HDAC inhibitor, DNA damaging platinum, or microtubule inhibitor class, also tend to be sensitive to other agents in that same class.

There are notable exceptions in the compound clusters. Interestingly, BEZ235 a dual ATP-competitive PI3K and mTOR inhibitor for p110α/γ/δ/β and mTOR (p70S6K) clustered away from the PI3K pathway inhibitors group and closer to a more diverse cluster containing MAPK/ERK pathway inhibitors and antimetabolites which are pathways strongly interconnected with mTOR signaling. Additionally, Nutlin-3a (a MDM-2 mediated stabilizer of tumor suppressor p53) clustered with a group on PI3K pathway inhibitors. Akt signaling is known to engage in control of MDM-2 mediated p53 levels and therefore the agent’s clustering highlights the similarities in underlying mechanism of action. These data suggest out-of-class treatment choices for cancers that have responded to an agent in the dominant class defining the cluster.

### Knowledge of intrinsic/prior drug response predicts response to over half of the drug panel

**Fig. 3:**
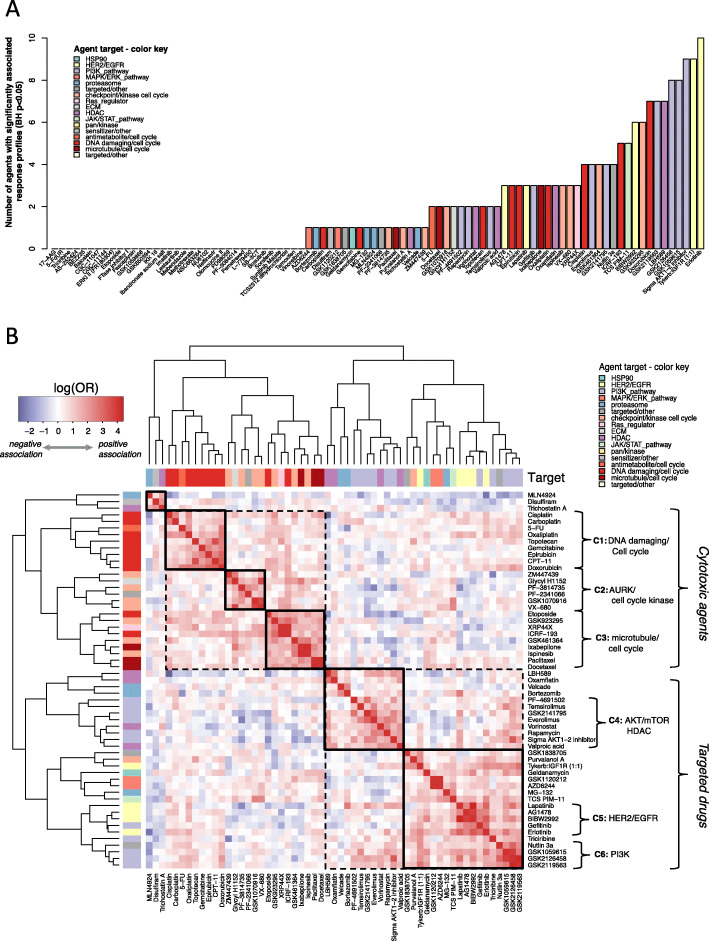
Prior/Intrinsic drug response associations. **a** Waterfall plot showing the number of agents with response that can be predicted (y axis; Fisher test BH *p* < 0.05) using knowledge of (prior/intrinsic) response of an agent (x axis). **b** Heatmap showing the odds ratios (log (OR))) of responding to one drug given knowledge of (intrinsic/prior) response of another drug, for all drugs in the panel with at least one significant predictive pair (BH p < 0.05). Red: positive association between drugs A and B (OR > 1; (intrinsic/prior) sensitivity to drug A predicts sensitivity to drug B, and (intrinsic/prior) resistance to A predicts resistance to B); Blue: negative association between drugs A and B (OR < 1; (intrinsic/prior) resistance to drug A predicts sensitivity to drug B, and vice versa). Two main clusters, cytotoxic vs. targeted agents, resolve into six main response clusters: (C1) DNA damaging/cell cycle, (C2) Aurora A kinase and cell cycle checkpoint inhibitors; (C3) microtubule poisons; (C4) AKT/mTOR/HDAC inhibitor mixed cluster; (C5) HER2/EGFR inhibitors; and (C6) PIK3C catalytic subunit inhibitors. Color bars represent manually annotated generalized modes of action. Clustering was performed using complete linkage and Pearson correlation distance

To more quantitatively address the question of which pairs or groups of agents have statistically significantly associated response profiles, we applied Fisher’s exact test to all pairs of agents (Fig. 3, Supplemental Figure SI2). If two agents are significantly associated, knowledge of (intrinsic/prior) sensitivity or resistance to one of the agents could in principle be used to improve the prediction of sensitivity to the other drug on the panel. We refer to this relationship as conditional sensitivity, i.e. response status to one compound associated with sensitivity/resistance to another agent.

After adjusting *p*-values for multiple testing, response to 57/90 drugs is significantly associated to response to at least one other agent (Fig. 3a). The number of associated drug response-prediction partners per drug ranged from 0 to 11, with the most highly connected nodes being erlotinib, tykerb: IGF1R, and an AKT1–2 inhibitor from Sigma, likely reflecting the enrichment for PI3K pathway inhibitors on the panel (Supplemental Table 1B).

We identified 33 compounds that did not exhibit conditional sensitivity; predominantly comprised of non-specific antimetabolite drugs and MAPK/ERK inhibitors. The antimetabolite compounds that fall into this category were either highly toxic to most cell lines irrespective of sensitivity profile (e.g., methotrexate), or relatively non-toxic (e.g., ibandronate). Interestingly, when investigating the complete set of compounds belonging to both drug classes, the therapeutics that did have a partner significantly associated with response – only had a single one, e.g. MEK1/2 inhibitors AZD6244 and GSK1120212 have only one significantly associated drug partner.

### Nearly all significant associations predict sensitivity to different agents in the same or a similar class

The heatmap in Fig. 3b shows the odds ratios (log (OR)) of responding to one drug given knowledge of (intrinsic/prior) response to another drug, for all drugs in the panel with at least one significant predictive pair (BH *p* < 0.05).

As described in Methods, *we use this OR matrix as a model for history-dependent response data*, in the absence of a dataset derived from cells (or patients) treated sequentially with multiple treatments. This model assumes (1) past response to a drug, whether sensitive or resistant, is based on *intrinsic rather than acquired* characteristics and (2) a cell line/patient’s intrinsic sensitivity/resistance profile remains unchanged despite the passage of time and treatment, at the time of the next decision point.

In the heatmap red represents a positive association (OR > 1), or similar pattern of response between agents. If an association is positive, (intrinsic/prior) sensitivity to one drug predicts sensitivity to a second drug; likewise, resistance to the first drug predicts resistance to the second drug. For example, (intrinsic/prior) sensitivity to carboplatin (column) predicts sensitivity to cisplatin (row, red). Overall, the clusters of response-associated agents fall into the broad classes of cytotoxic vs. targeted drugs (Fig. 3b). Within the larger cytotoxic cluster are three subclusters: (C1) commonly used DNA damaging agents (e.g., platinums and anthracyclines), (C2) cell cycle kinase inhibitors (e.g., VX-680), and (C3) microtubule poisons including taxanes and ixabepsilone. The targeted agent group contains smaller clusters of response-associated agents featuring (C5) HER2/EGFR inhibitors; (C6) PI3K inhibitors; and (C4) a mixed group of AKT/mTOR/HDAC inhibitors (Fig. 3b). Multiscale bootstrap resampling performed to estimate *p*-values for hierarchical clustering established clusters (C1-C3 and C5-C6) to be highly supported by the data with p-values < 0.05 (Supplemental Figure S3). Cell lines sensitive to one agent in any of these clusters are likely to be sensitive to others in the same cluster. Mixed cluster C4 was not highly supported but there are drug pairs within the cluster, such as rapamycin and vorinostat, with significantly associated responses BH *p* < 0.05 (Supplemental Figure S2B).

Associations that might be less apparent in a clustered response heatmap such as Fig. 2 are of the ‘cross response type’ variety, wherein (intrinsic/prior) resistance to one drug predicts sensitivity to a different drug or vice versa. This class of relationship is of special interest, because of its potential utility in identifying an effective next treatment for patients who were highly resistant to a prior therapy. However, of the 88 significant drug pair response associations (BH Fisher *p* < 0.05), 85 represent similar profiles of response; and only 3 represent cross-response class associations (Supplemental Figure S4). These resistance-conditioned sensitivity pairs are alkylating antineoplastic agent cisplatin and an HDAC inhibitor LBH589; EGFR inhibitor erlotinib and neddylation inhibitor MLN4924, and anti-helminthic mebendazole and cell cycle inhibitor purvalanol A.

### Recommendation system built on in vitro data of drug response further reveals conditional patterns of drug response in breast cancer cell lines

In order to further refine history dependent response predictions for breast cancer cell lines, as well as create a list of the top recommended compounds, we utilized a recommender systems analysis as detailed in the Methods section. Applying this method to our binary response matrix we obtained a dataset with 200 non-redundant probability of drug response rules with a minimum confidence level of 0.8 and a minimum support of 0.2. Only rules with a one-to-one drug relationship were considered. For each rule, a lift value was calculated to indicate the strength of association between the antecedent drugs (LHS) and the consequent drug recommendations (RHS). This analysis enables us not only to investigate drug associations, but also consider four different types of associations: (1) if sensitive to drug A, then sensitive to drug B (discovered associations *n* = 92), (2) if sensitive to drug A, then resistant to drug B (*n* = 25), (3) if resistant to drug A, then also resistant to drug B (*n* = 70), and (4) if resistant to drug A then sensitive to drug B (*n* = 13).

**Fig. 4:**
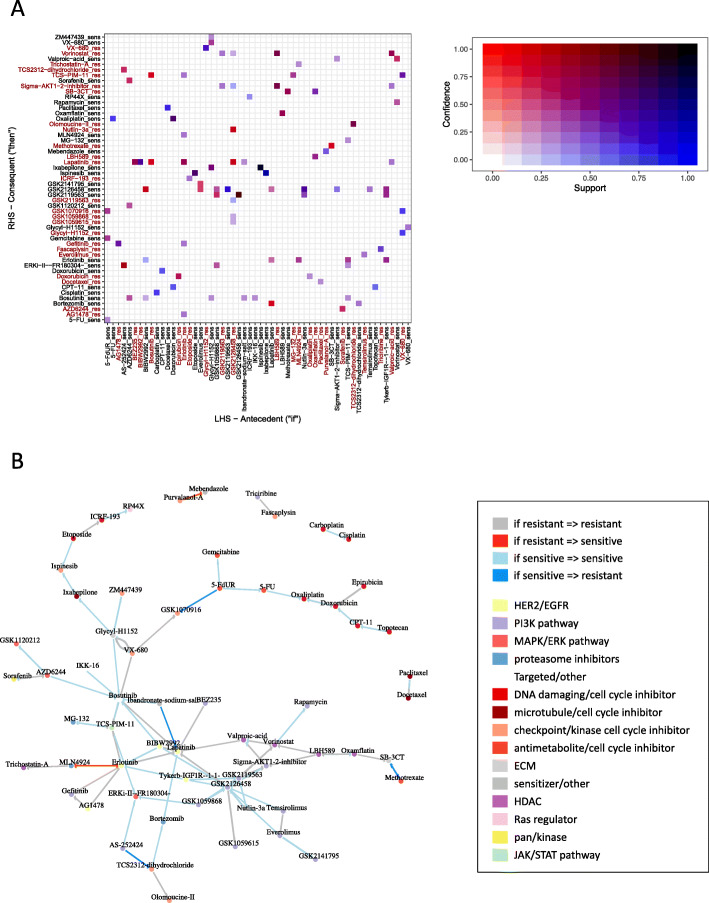
Recommender system results. **a** Graph representation of 100 rules with highest lift values; vertices represent drugs and are colored to reflect drug target classification as specified in the legend. Edges indicate the rule-governed relationships between them, colored based on the type of relationship. **b** Matrix plot of all discovered rules that met the 0.2 support and 0.8 confidence cutoff criteria. Squares are colored by combination of confidence and support values with more red representing higher confidence in the association, with blue representing higher support for the association. Axis labels were colored to help identify drug relationships: dark red corresponding to “resistant” and grey corresponding to “sensitive”. In addition, “_sens” and “_res” types of relationships were appended to the drug names in axis labels and correspond to “sensitive” and “resistant” respectively

The mined rules were used to create a directional network of dependencies between drugs, presumably mediated by relationships between mechanisms of action and the spectrum of therapeutic vulnerabilities in breast cancer. The high complexity of such a representation is better visualized over smaller subsets of the graph; therefore, the graph visualization in Fig. 4a highlights the hundred strongest (highest lift) association rules. A complete list of mined rules is further provided in Supplemental Table 2. The arrows in the plot correspond with association type and begin from a label representing an observed antecedent (LHS, intrinsic) drug response (resistance or sensitivity), and point towards the consequent (RHS) drug response. For example, the strongest association according to this analysis is (lift = 2.262) supports an association between sensitivity to HER2/EGFR inhibitor Lapatinib (LHS), and consequent sensitivity to Erlotinib – another EGFR inhibitor (RHS).

The findings made by recommender system around sensitive-to-sensitive relationships, mirror closely observations from previous OR-based clustering analyses. All of the previously described drug categories (C1-C6, Fig. 3b) can be traced back to node clusters in the recommender system graph (Fig. 4a). For example, we can see proximal connectivity, i.e. a shared pattern of conditional drug response, between groups of DNA damaging/cell cycle inhibitors like Carboplatin and Cisplatin, as well was Oxaliplatin, Doxorubicin, CPT-11, Epirubicin and Topotecan, mirroring findings of Cluster (C1), as well as PI3K pathway inhibitors found in group (C6) (Fig. 3b), i.e. GSK2119563, GSK2126458, GSK1059615, Everolimus, Temsirolimus and GSK2141795.

There are a number of new findings in this analysis that are not observed with the Odds ratio model approach, and they often revolve around associations with opposite response types (“if resistant, then sensitive” and “if sensitive then resistant”). These types of recommendations would be most valuable in clinical settings when next lines of treatment for patients with drug resistance. The recommender system analysis revealed thirteen associations of this specific type, the strongest being the relationships between HER2 inhibitor Erlotinib and proteasome inhibitor MLN4924. In this case, the recommender system suggests Erlotinib for MLN4924 resistant cell lines (lift = 2.07, confidence = 0.857) and vice versa, MLN4924 for lines resistant to Erlotinib (lift = 2.0454, confidence = 0.818). Interestingly, the system also recommends a proteasome inhibitor Bortezomib if intrinsic sensitivity to Lapatinib (HER2 inhibitor) was detected (confidence = 0.875, lift = 2.041) – an association not revealed in prior analysis (Fig. 3b Fisher BH *p* = 0.076).

### Conditional patterns of drug response in known breast cancer molecular subtypes

A natural question is whether agent-response dependencies vary by molecular subtype. Though numbers are very small, we used the recommendation system side by side with the OR matrix approach to explore differences in drug response patterns in the three select molecular subtypes represented in the dataset: luminal (*n* = 17), ERBB2-amplified (*n* = 10), and basal or claudin-low combined (*n* = 18) (Supplemental Figure 5). After correcting for multiple hypothesis testing of the OR matrix, we found significantly similar response profiles for two compounds in the luminal set, six in basal or claudin low, and none in the smallest ERBB2-amp group (Supplemental Figure 5A). In the basal and claudin-low group we see clustering of docetaxel and paclitaxel, both chemotherapeutic taxanes, as well as of PI3K-targetting drugs GSK2126458 and GSK2119563, closely related in mechanism of action to a nearby MEK inhibitor AZD6244. It is noteworthy that the recommender system highlighted these same relationships in each grouping, when sorted by top lift scores (Supplemental Figure 5B, C), but also includes additional compounds within the drug cluster, e.g., docetaxel and paclitaxel are joined by another checkpoint inhibitor, vinorelbine. In addition, the recommender system captures heterogeneous response edge types, e.g., in the luminal subtype, cells that are resistant to Olomucine II, are likely to be sensitive to ERKi II (FR180304). While our conclusions are limited by small subset size, this analysis suggests the consistency of findings as well as potential utility for applying history-dependent response quantification in a molecular subtype context.

## Discussion

Typically, managing metastatic breast cancer care involves selecting multiple drugs regimens over patient’s lifetime, with new therapeutics being introduced sequentially upon progression or in the case of accumulated toxicity. Though information regarding response to past treatments may provide clues regarding the classes of drugs most and least likely to work for a particular patient, currently there is no systematized knowledge base that would support clinical treatment decision-making that takes past treatments and responses into account. Such a knowledge base could also be useful for emerging neoadjuvant clinical trial designs in early stage breast cancer that adapt within individual patients using response data from the first treatment block to help select the second treatment block, with the goal of maximizing the probability of achieving a pCR.

Ideally, to address these needs one would develop a history-dependent drug response predictor based on data from cells (or patients) treated sequentially with multiple treatments. Lacking such data, here we propose a *odds ratio (log (OR)) matrix as a model for history-dependent response data.* Briefly, to generate this matrix we analyzed previously published in vitro viability data obtained from 91 breast cancer cell lines. We calculated the OR of response to a variety of compounds given information on sensitivity or resistance to other agents using an in vitro cell line drug screen panel. We thus identified the subset of 57 compounds with a response that can be predicted by a ‘history’ of either sensitivity or resistance to one or more of the 90 drugs on the panel, as well as the most effective follow-on treatments for each specific drug. Utilizing clustering methods after filtering for significance, we established five highly data supported clusters of compounds with shared response patterns: (C1) commonly used DNA damaging agents (e.g., platinums and anthracyclines), (C2) cell cycle kinase inhibitors (e.g., VX-680), (C3) microtubule poisons including taxanes and ixabepsilone, (C4) a mixed group of AKT/mTOR/HDAC inhibitors, (C5) HER2/EGFR inhibitors and (C6) PI3K inhibitors. The similar patterns of response may help guide selection of alternative drug options for individual patients; and in addition, may help elucidate the mechanisms of action for each compound and the relationships between agents.

Though we hoped to identify pairs and groups of agents with cross-response relationships, wherein (intrinsic/prior) resistance to one drug predicts sensitivity to one or more agents, our analysis uncovered few results of this type. Rather, nearly all the significant response-prediction agent pairs were for similar response profiles, which could be useful to help prioritize a ‘next’ treatment for patients who initially responded to an agent, perhaps prior to one or more intervening treatments of a different class. This type of approach is already widely used in HER2+ and ER+ metastatic breast cancer, where patients are treated with a series of different anti-HER2 or endocrine agents, respectively, resulting in longer survival times. This project begins to establish a rational basis for inclusion of additional agents for these patients, and supports a similar strategy for other subtypes and agent classes.

In addition to the approach of applying hierarchical clustering to an OR matrix model of history-dependent response data, we employed data mining algorithms that use drug response ‘history’ to further inform the best strategies to circumvent treatment resistance. Frequent item set mining and association rule induction [15, 16] are powerful methods for so-called market basket analysis, which aims at finding regularities in the shopping behavior of customers of supermarkets, online shops etc. With the induction of frequent item sets and association rules one tries to find sets of products that are frequently bought together, so that from the presence of certain products in a shopping cart one can infer (with a high probability) that certain other products are present. Such information, especially if expressed in the form of association rules, can often be used to increase the number of items sold, for instance, by directly suggesting items to a customer. We treated sensitivity and resistance responses to drugs similarly to shopping transactions, and breast cancer cell lines, to customers. Through this approach we were able to find and examine four types of associations between directional response patterns: (1) if sensitive to drug A, then sensitive to drug B, (2) if sensitive to drug A, then resistant to drug B, (3) if resistant to drug A, then also resistant to drug B, and (4) if resistant to drug A then sensitive to drug B which further confirmed our Odds-ratio-model-based findings, as well as established new ones such as sensitivity to proteasome inhibitor Bortezomib if intrinsic sensitivity to Lapatinib (HER2 inhibitor) was detected (confidence = 0.875, lift = 2.041) – an association not revealed in prior analysis (Fisher BH *p* = 0.076). The increased observational power could be attributed to the directionality of recommender analysis, which is not available in the symmetrical approach of a Fisher’s exact test.

The utilization of these approaches comes with certain limitations. On a fundamental level, the first limitation is in assuming that cancer cell lines studied in vitro are reasonable models of tumors in patients, and that drug response can be fully determined by GI50 threshold values from in vitro viability studies. Evaluation of cancer therapeutics where cytotoxicity is not a primary mode of action may become less informative, for example ibandronate sodium salt, a bone resorption blocking activity meant to prevent metastasis rather than kill tumor cells. This problem could be systematically addressed by excluding non-cytotoxic compounds, restricting viability data to clinically relevant combinations of compounds, or/and including other drug response measures such as apoptotic activity or cell cycle arrest. Also, more realistic models such as xenografts or mouse models in immune-intact mice could be employed.

Another fundamental issue arises from the fact that once tumors are treated, their biology changes, though the maintenance of basic pathways of intrinsic sensitivity/resistance forms the basis for (mostly successfully) treating HER2+ metastatic patients with sequential HER2-targeted agents, and likewise for treating ER+ metastatic patients with sequential hormone modulators. The recommendations created from non-sequential treatment data, while helpful for patients with cancers that maintain basic driving mechanisms of intrinsic sensitivity/resistance, are likely to be deficient in addressing acquired resistance that substantially modifies tumor drivers. To amend this limitation, additional data from sequential treatment in vitro viability panels are necessary, or better yet longitudinal data on treatment, response, and response duration from a large number of metastatic patients and/or early stage patients treated with sequential neoadjuvant therapies. Utilizing sequential treatment data, one could explore temporal dependencies, i.e. if a patient had a good response to drug A in the past, but then eventually progressed and has since been through three more lines of treatment, is drug A likely to work again? These data could be used to construct an OR model as well, though with ‘real’ history-conditioned sensitivity experiments the matrix would likely be asymmetric, if treatment order modulates the probability of response.

With the recent advancement of technology and increased knowledge of transcriptional subtypes in breast cancer, it will also be essential to include this type of information in the recommendation systems. In order to avoid over-fitting of such predictions, larger datasets than the one studied here are required. Increasing the amount of data will also provide more support for each recommendation making the predictions even more reliable.

Not every path in breast cancer therapy is straightforward, and clinicians face difficult decisions every day. The use of big biomedical datasets and data mining methods such as recommendation systems, could be pivotal in informing clinicians deciding on next line of breast cancer treatments for treatment-resistant or progressing patients.

## Conclusions

In this manuscript we propose a rational, history-dependent, data-supported analytical approach for cancer treatment selection based on prior drug sensitivity. Our findings from the association statistical analysis support a strategy of treating patients with different agents in the same class where sensitivity was previously observed. The directionality of complementary recommender analysis allows for discovery of additional drug associations where resistance to prior treatment corresponded with sensitivity to another compound. We believe that the presented approach may be pivotal in informing clinicians deciding on the next line of treatments for patients who have progressed on their current regimen.

## Supplementary Information

**Additional file 1: Supplemental Figure S1.** Approximately Unbiased (AU) and Bootstrap Probability (BP) *p*-values for hierarchical clustering of the binarized response dataset (Fig. 2). Based on multiscale bootstrap resampling, clusters that are highly supported by the data will have large AU values. Blue squares (AU = 0.9) and red lines (AU = 0.95) represent clusters highly supported by the data. **Supplemental Figure S2.** A) Waterfall plot showing the number of agents with response that can be predicted (y axis; Fisher test, uncorrected *p* < 0.05) using knowledge of (prior/intrinsic) response of an agent (x axis). B) Heatmap showing the odds ratios (log (OR))) of responding to one drug given knowledge of (intrinsic/prior) response of another drug, for only those drug pairs that are significantly associated (uncorrected p < 0.05). Red: positive association between drugs A and B (OR > 1, p < 0.05; (intrinsic/prior) sensitivity to drug A predicts sensitivity to drug B, and (intrinsic/prior) resistance to A predicts resistance to B); Blue: negative association between drugs A and B (OR < 1, *p* < 0.05; (intrinsic/prior) resistance to drug A predicts sensitivity to drug B, and vice versa). White squares denote pairs with *p* > 0.05. **Supplemental Figure S3.** Approximately Unbiased (AU) and Bootstrap Probability (BP) *p*-values for hierarchical clustering of the OR matrix model of history-dependent response (Fig. 3b). Based on multiscale bootstrap resampling, clusters that are highly supported by the data will have large AU values. Blue squares (AU = 0.9) and red lines (AU = 0.95) represent clusters highly supported by the data. **Supplemental Figure S4.** Heatmap showing the odds ratios (log (OR)) of responding to one drug given knowledge of (intrinsic/prior) response of another drug, for only those drug pairs that are significantly associated after multiple testing correction (BH *p* < 0.05). Red: positive association between drugs A and B (OR > 1, BH p < 0.05; (intrinsic/prior) sensitivity to drug A predicts sensitivity to drug B, and (intrinsic/prior) resistance to A predicts resistance to B); Blue: negative association between drugs A and B (OR < 1, BH *p* < 0.05; (intrinsic/prior) resistance to drug A predicts sensitivity to drug B, and vice versa). White squares denote pairs with BH *p* > 0.05. **Supplemental Figure S5**. A) Heatmap showing the odds ratios (log (OR))) of responding to one drug given knowledge of (intrinsic/prior) response of another drug in luminal (*n* = 17) and basal or claudin-low (*n* = 18), and ERBB2-amp molecular subtypes (*n* = 10). Red: positive association between drugs A and B (OR > 1, p < 0.05; (intrinsic/prior) sensitivity to drug A predicts sensitivity to drug B, and (intrinsic/prior) resistance to A predicts resistance to B); Blue: negative association between drugs A and B (OR < 1, p < 0.05; (intrinsic/prior) resistance to drug A predicts sensitivity to drug B, and vice versa). White squares denote pairs with p > 0.05. Top panel: shown for all drugs (uncorrected p < 0.05), Bottom panel: shown for only those drug pairs that are significantly associated (BH p < 0.05). B) Graph representation of 100 rules with highest lift values for vertices represent drugs and are colored to reflect drug target classification as specified in the legend for subset of luminal (n = 17), basal or claudin-low (n = 18), and ERBB2-amp molecular subtypes (n = 10). Edges indicate the rule-governed relationships between them, colored based on the type of relationship: dark red corresponding to “resistant” and grey corresponding to “sensitive”. In addition, “_sens” and “_res” types of relationships were appended to the drug names in axis labels and correspond to “sensitive” and “resistant” respectively. C. Tables representing top 10 rules mined for each breast cancer molecular subtype, indicating detailed statistics including confidence, support, lift and edge type. (PPTX 9393 kb)

**Additional file 2: Supplemental Table 1.** A) Cell lines used in this publication [7, 17–19]; with their transcriptional subtype and ERBB2 status; B) List of compounds used to treat the breast cancer cell lines with their generalized target, and derived IQR response data.

**Additional file 3: Supplemental Table 2.** Complete list of recommender system rules with one-to-one drug relationships. “_sens” and “_res” types of relationships were appended to the drug names and correspond to “sensitive” and “resistant” respectively.

## Data Availability

All data generated or analyzed during this study were previously published (Daemen A, et al., Genome Biology. 2013;14:R110.)

## Abbreviations

OR: Odds ratios
TN: Triple negative
MBC: Metastatic breast cancer
ER: Estrogen receptor
HER2: Human epidermal growth factor receptor 2
pCR: Pathologic complete response
GI50: Growth inhibition by 50%
BH: Benjamini Hochberg
LHS: Left hand side
RHS: Right hand side
AU: Approximately unbiased
BP: Bootstrap probability
HDAC: Histone deacetylase
