# Supplementary material for: Toward developing a metastatic breast cancer treatment strategy that incorporates history of response to previous treatments
Source: BMC Cancer. 2021 Mar 1;21:212. doi: 10.1186/s12885-021-07912-7 (PMC7923477; doi:10.1186/s12885-021-07912-7)
Supplement: Supplementary file 1 — Additional file 1: Supplemental Figure S1. Approximately Unbiased (AU) and Bootstrap Probability (BP) p-values for hierarchical clustering of the binarized response dataset (Fig. 2). Based on multiscale bootstrap resampling, clusters that are highly supported by the data will have large AU values. Blue squares (AU = 0.9) and red lines (AU = 0.95) represent clusters highly supported by the data. Supplemental Figure S2. A) Waterfall plot showing the number of agents with response that can be predicted (y axis; Fisher test, uncorrected p < 0.05) using knowledge of (prior/intrinsic) response of an agent (x axis). B) Heatmap showing the odds ratios (log (OR))) of responding to one drug given knowledge of (intrinsic/prior) response of another drug, for only those drug pairs that are significantly associated (uncorrected p < 0.05). Red: positive association between drugs A and B (OR > 1, p < 0.05; (intrinsic/prior) sensitivity to drug A predicts sensitivity to drug B, and (intrinsic/prior) resistance to A predicts resistance to B); Blue: negative association between drugs A and B (OR < 1, p < 0.05; (intrinsic/prior) resistance to drug A predicts sensitivity to drug B, and vice versa). White squares denote pairs with p > 0.05. Supplemental Figure S3. Approximately Unbiased (AU) and Bootstrap Probability (BP) p-values for hierarchical clustering of the OR matrix model of history-dependent response (Fig. 3b). Based on multiscale bootstrap resampling, clusters that are highly supported by the data will have large AU values. Blue squares (AU = 0.9) and red lines (AU = 0.95) represent clusters highly supported by the data. Supplemental Figure S4. Heatmap showing the odds ratios (log (OR)) of responding to one drug given knowledge of (intrinsic/prior) response of another drug, for only those drug pairs that are significantly associated after multiple testing correction (BH p < 0.05). Red: positive association between drugs A and B (OR > 1, BH p < 0.05; (intrinsic/prior) sensitivi [file 12885_2021_7912_MOESM1_ESM.pptx]

## Slide 1
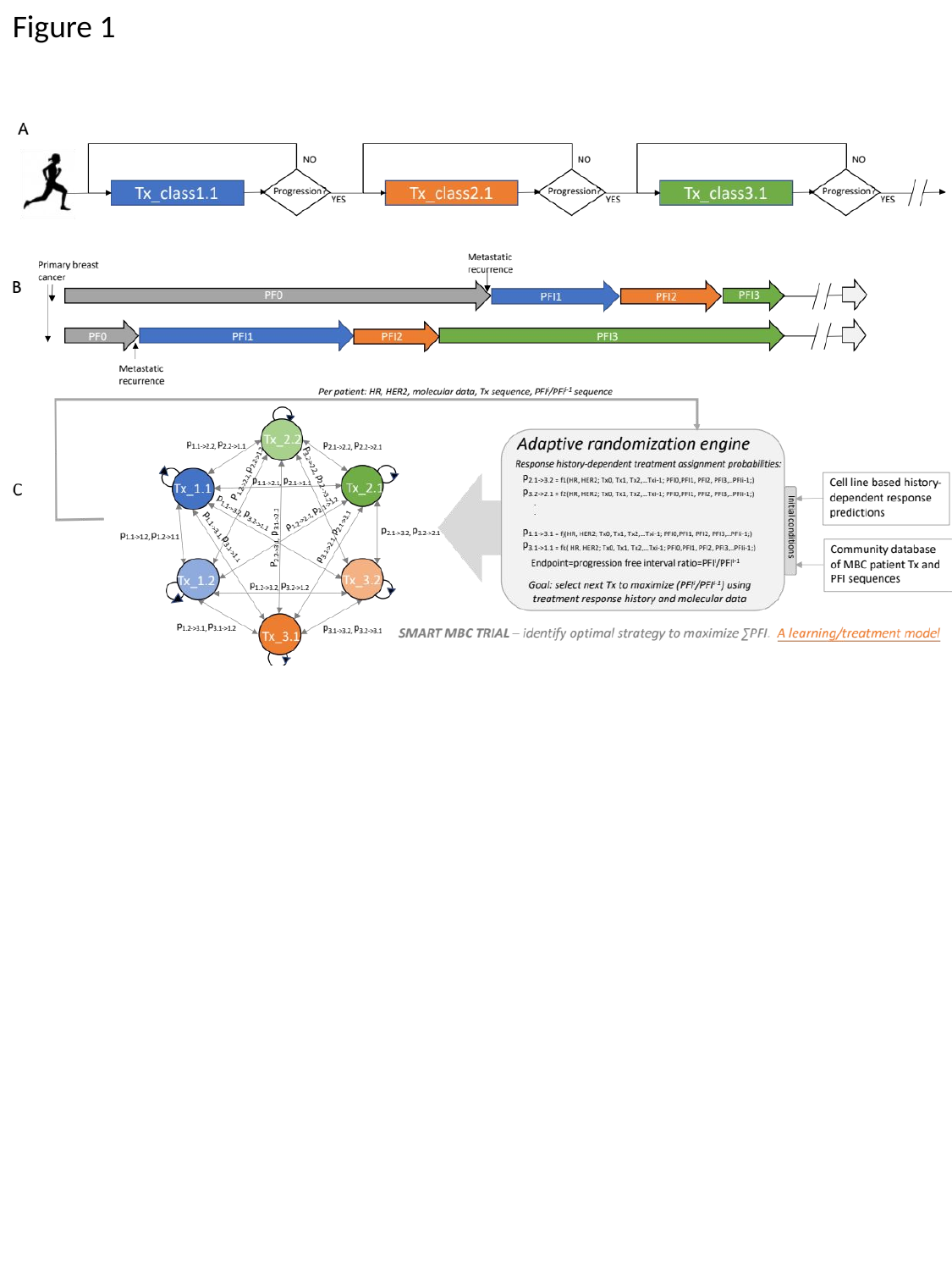

Figure 1

## Slide 2
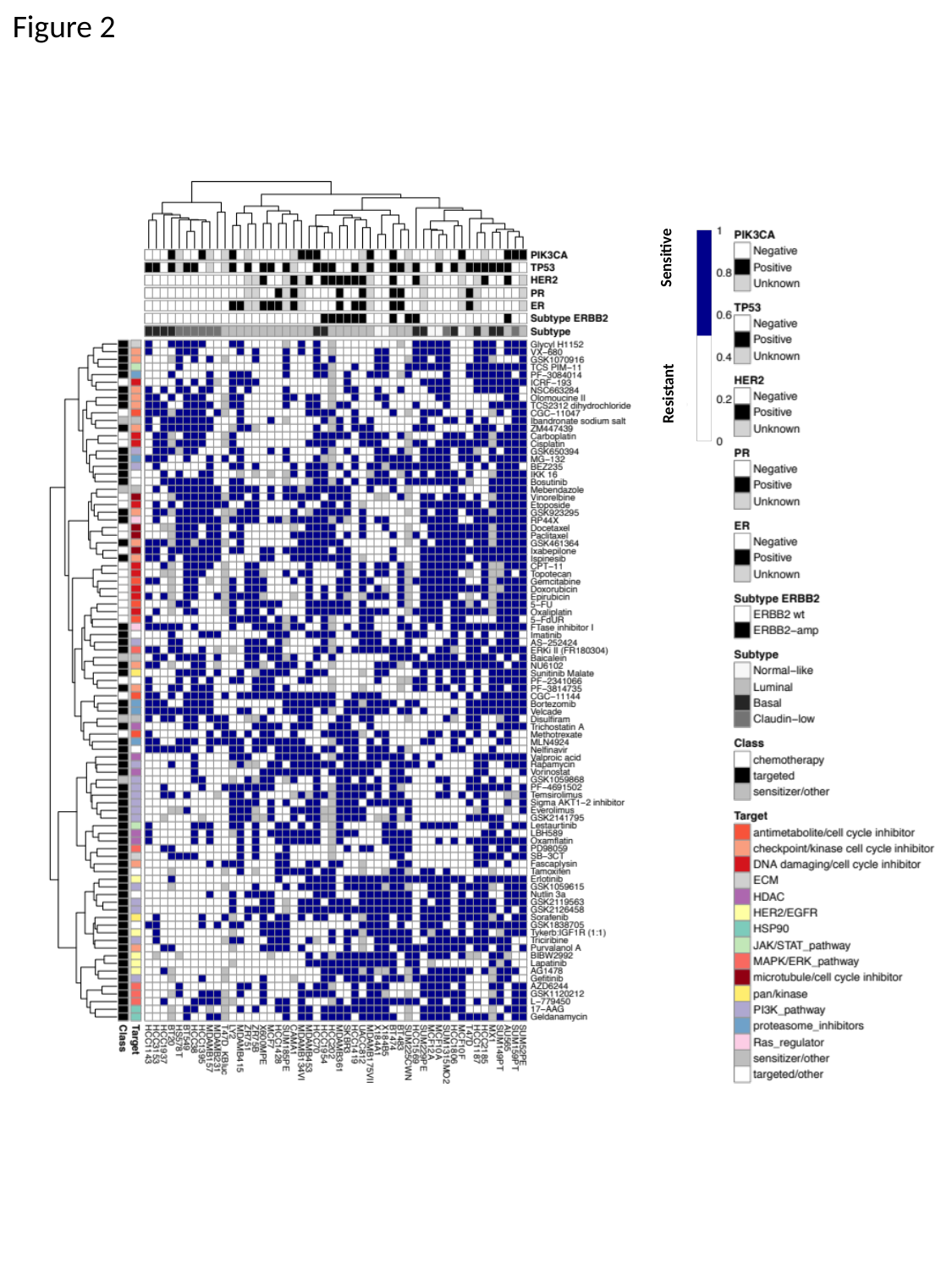

Figure 2
Sensitive
Resistant

## Slide 3
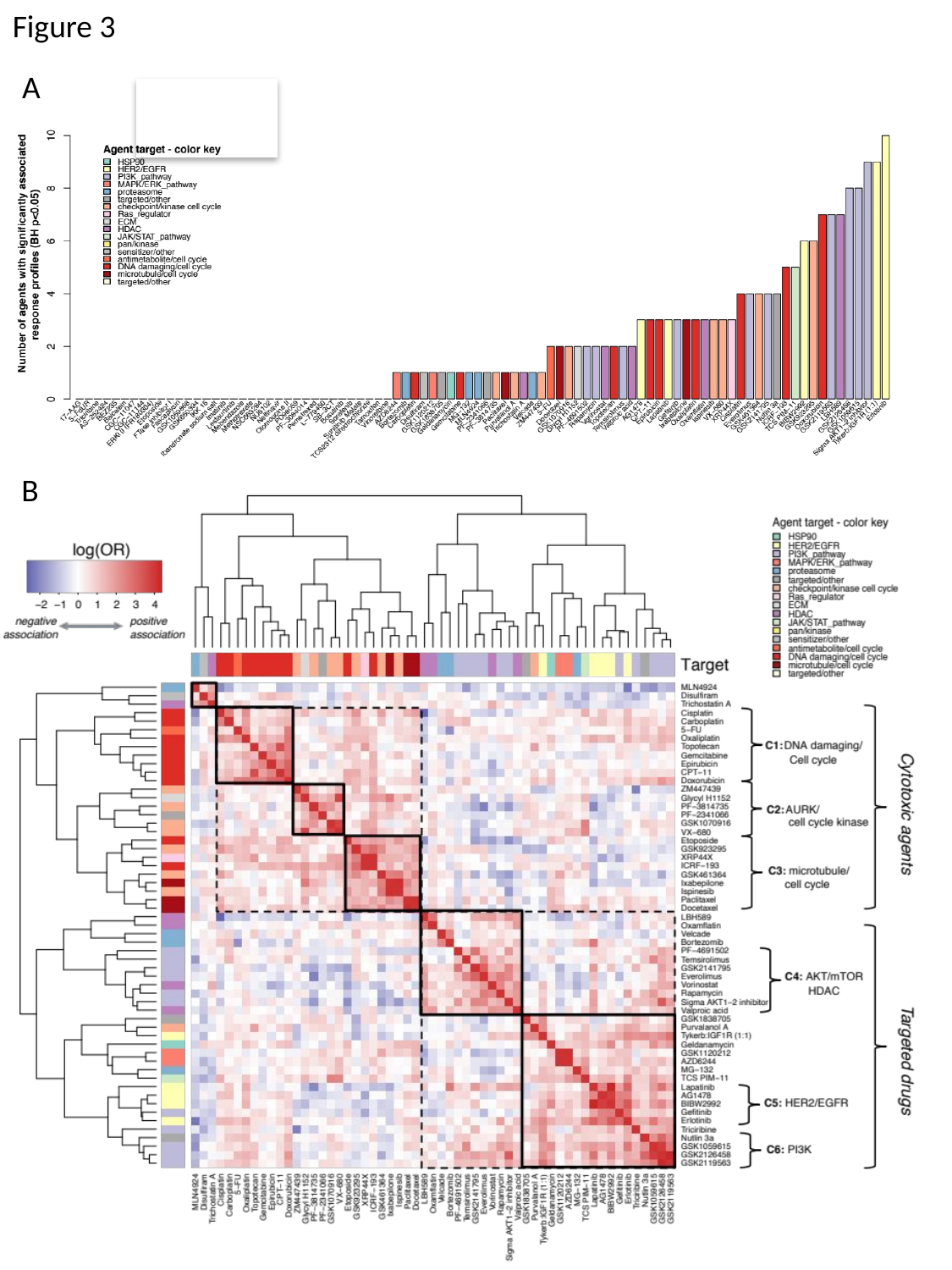

Figure 3
A
B

## Slide 4
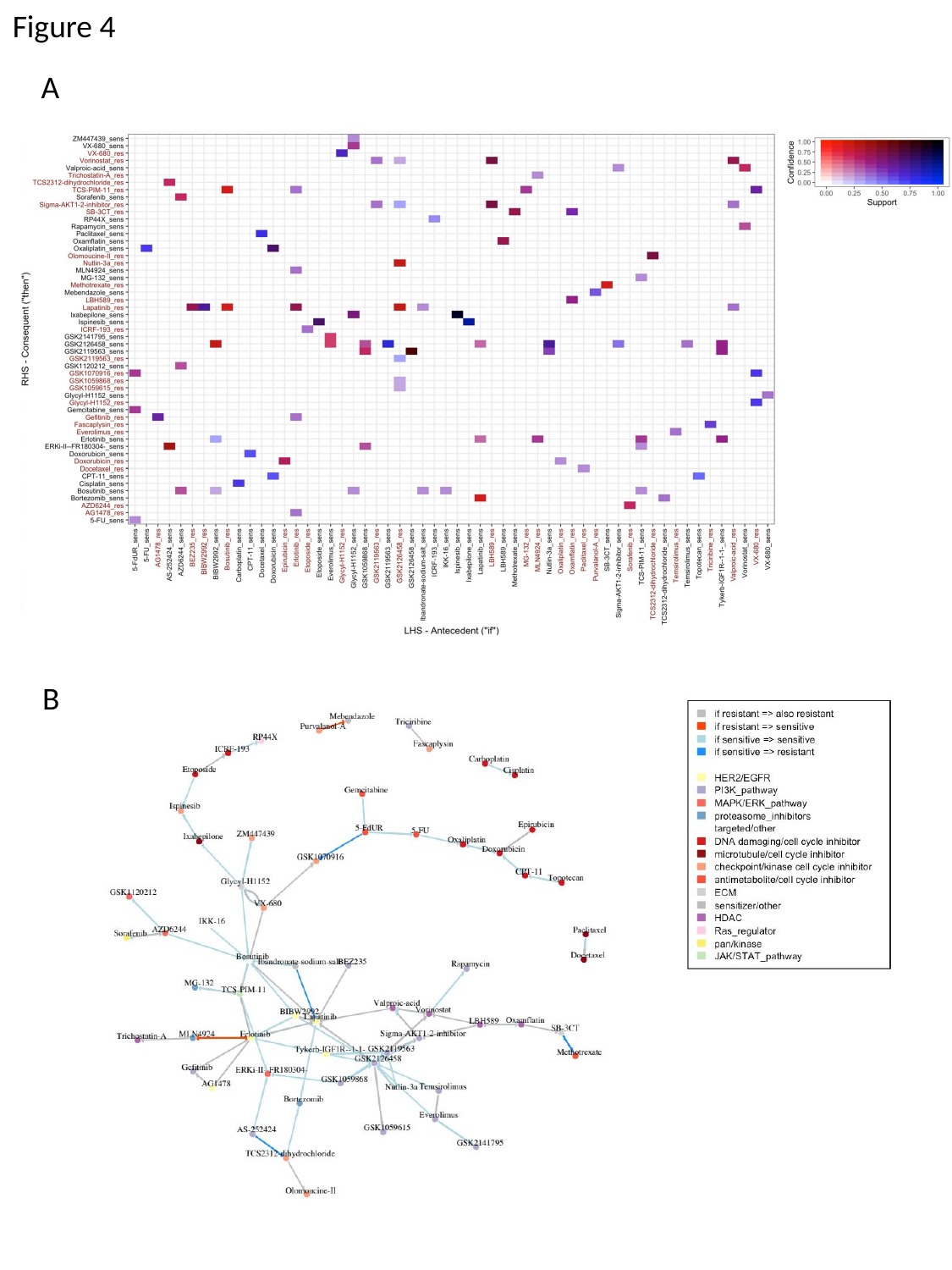

Figure 4
A
B

## Slide 5
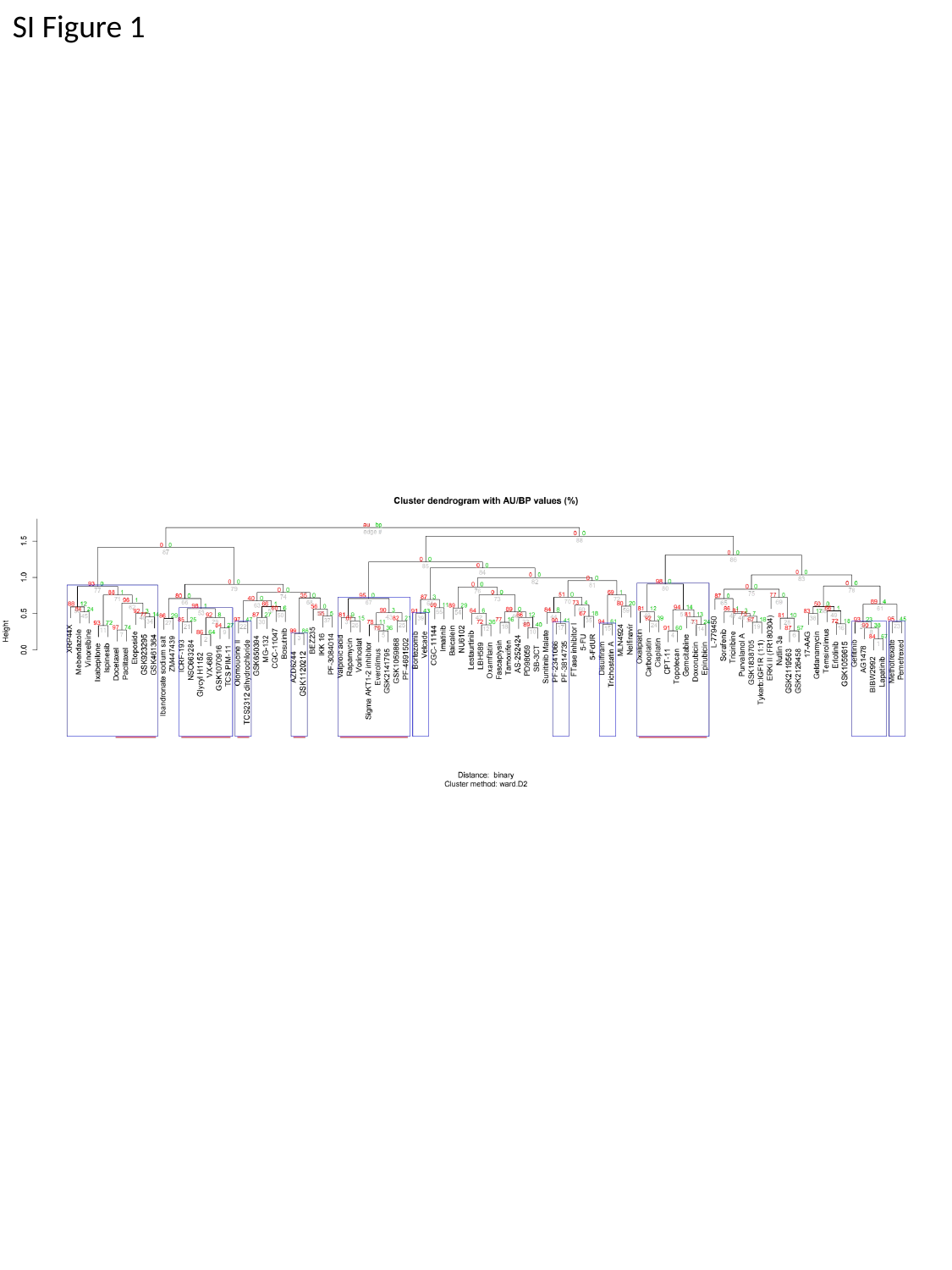

SI Figure 1

## Slide 6
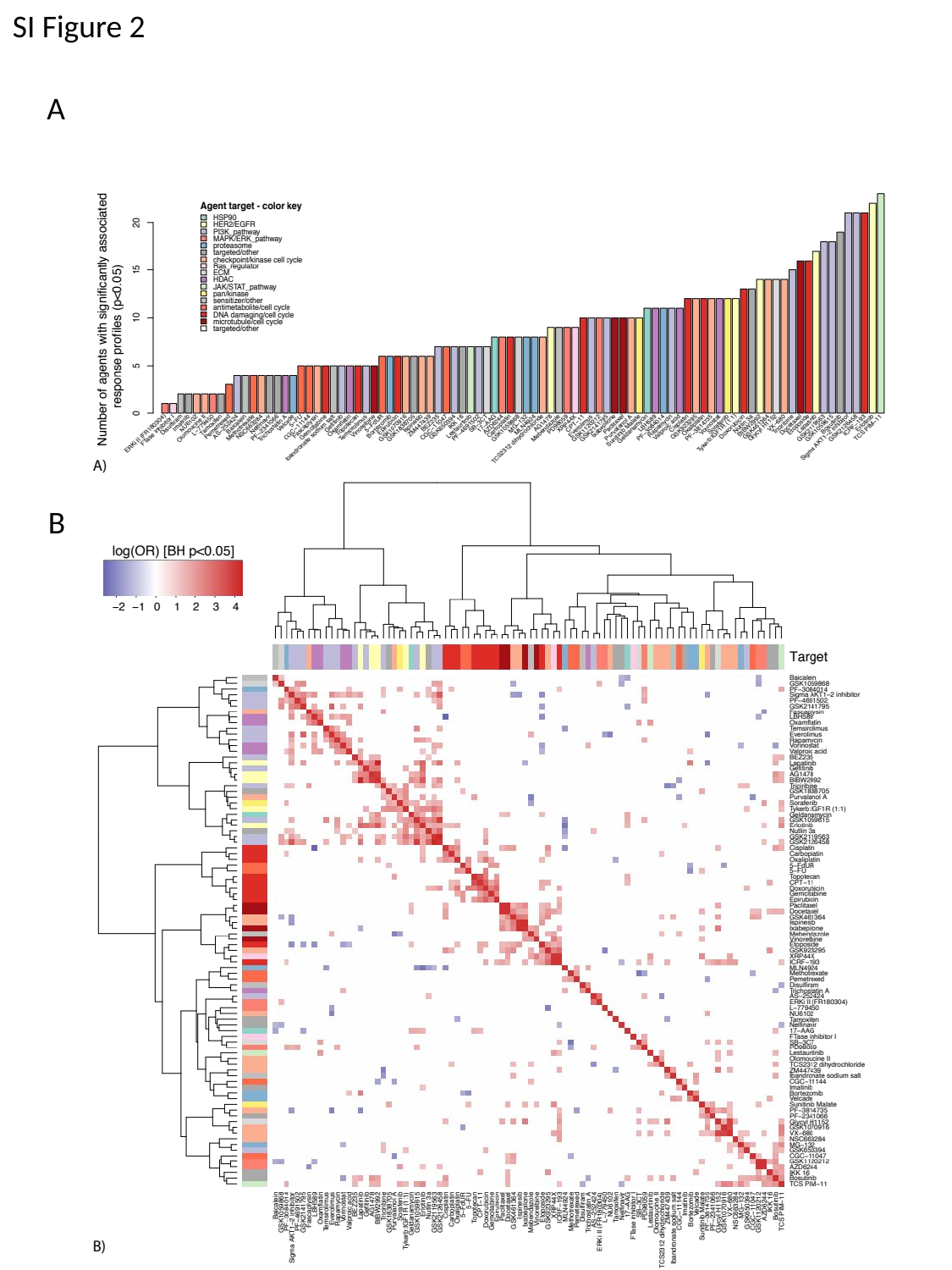

SI Figure 2
A
B

## Slide 7
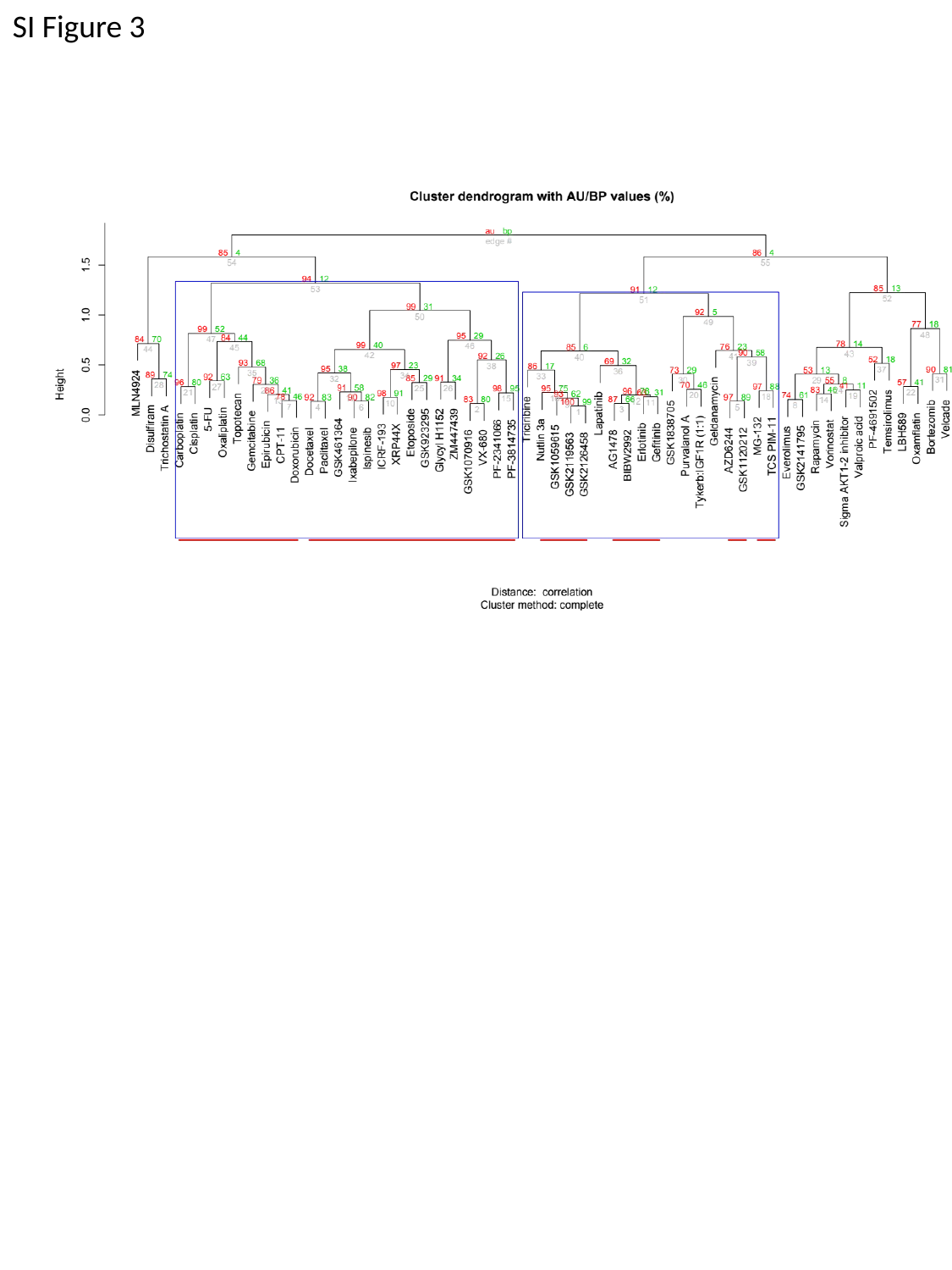

SI Figure 3

## Slide 8
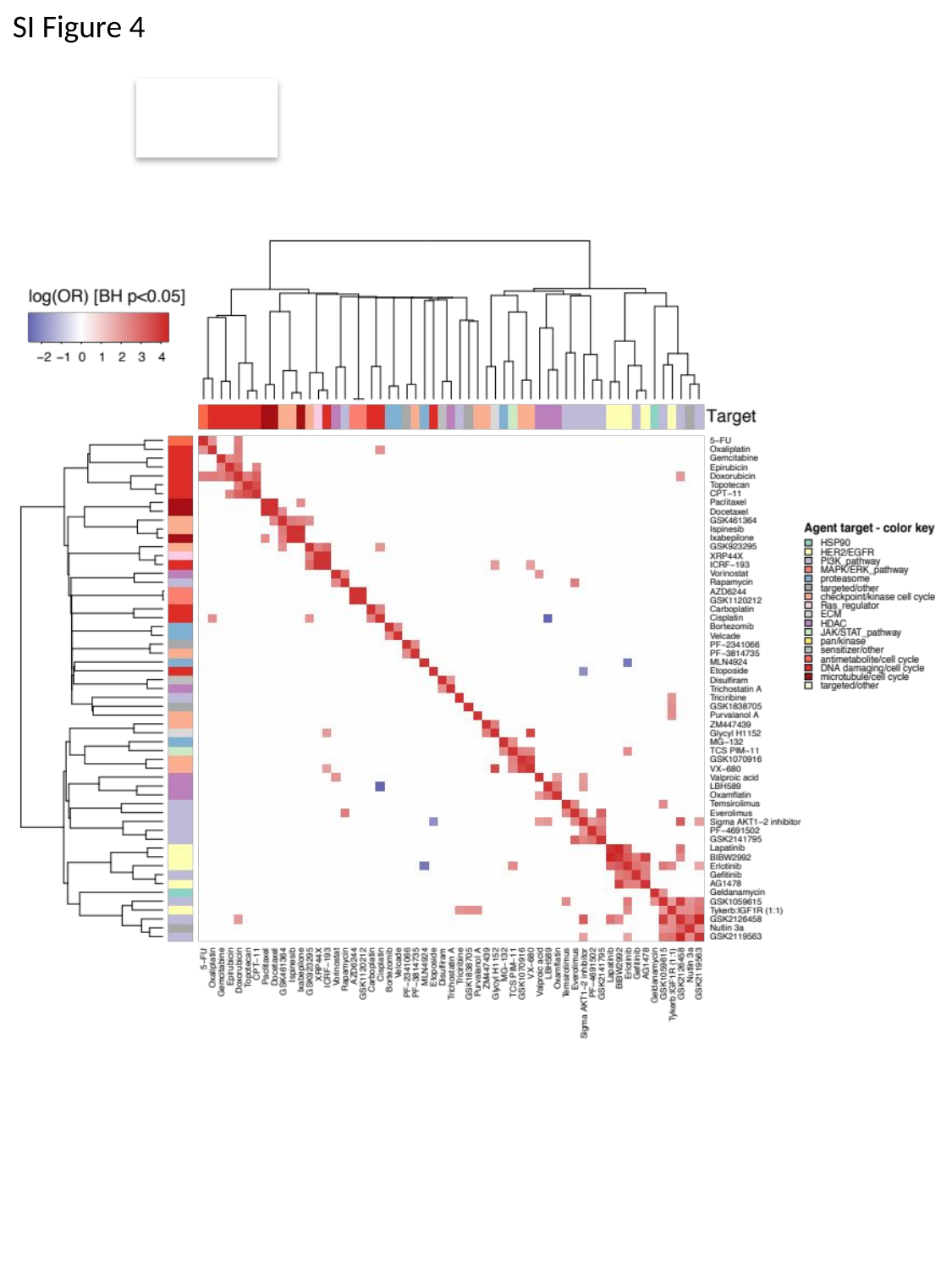

SI Figure 4

## Slide 9
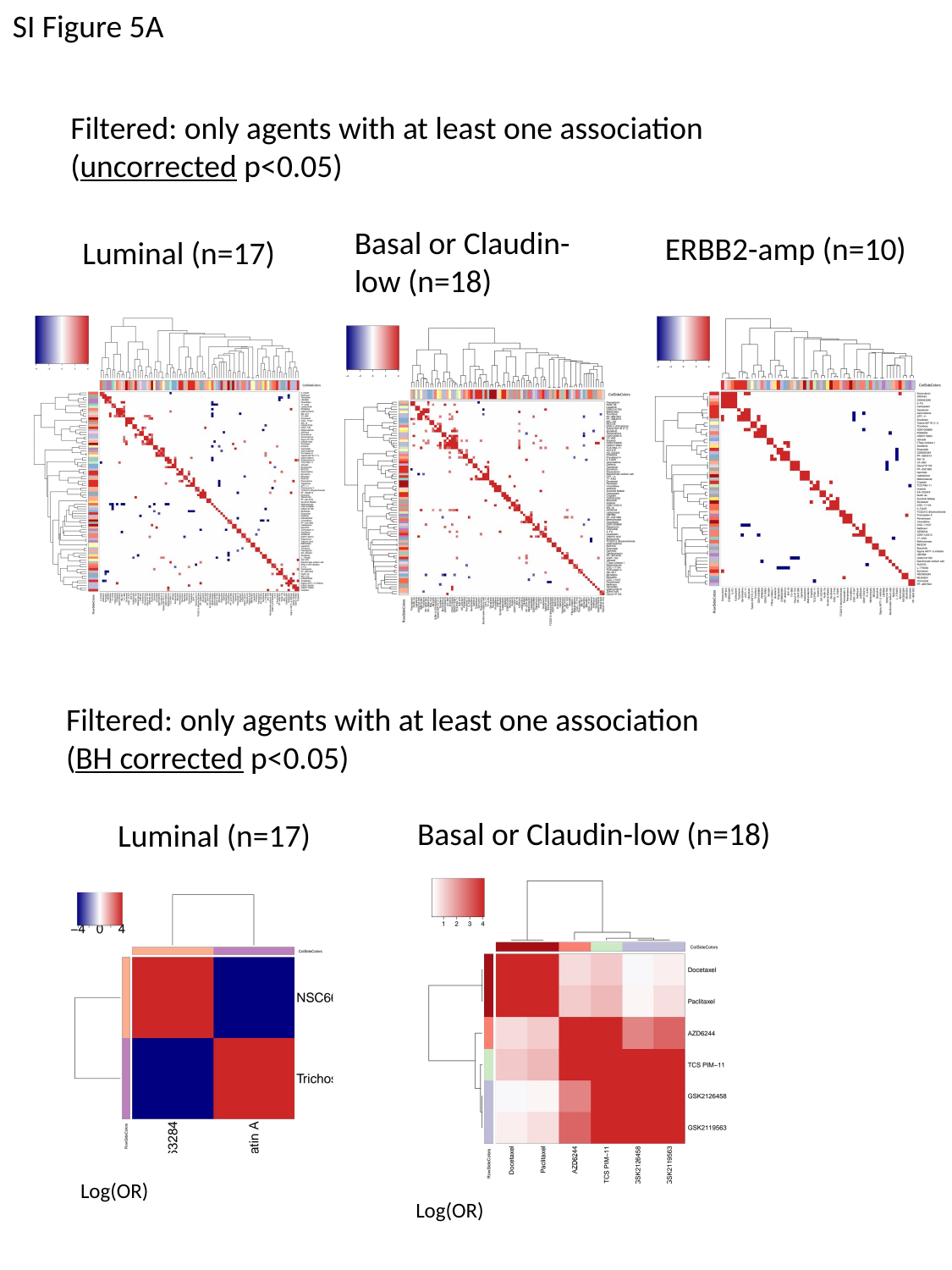

SI Figure 5A
Filtered: only agents with at least one association (uncorrected p<0.05)
Basal or Claudin-low (n=18)
ERBB2-amp (n=10)
Luminal (n=17)
Filtered: only agents with at least one association (BH corrected p<0.05)
Basal or Claudin-low (n=18)
Luminal (n=17)
Log(OR)
Log(OR)

## Slide 10
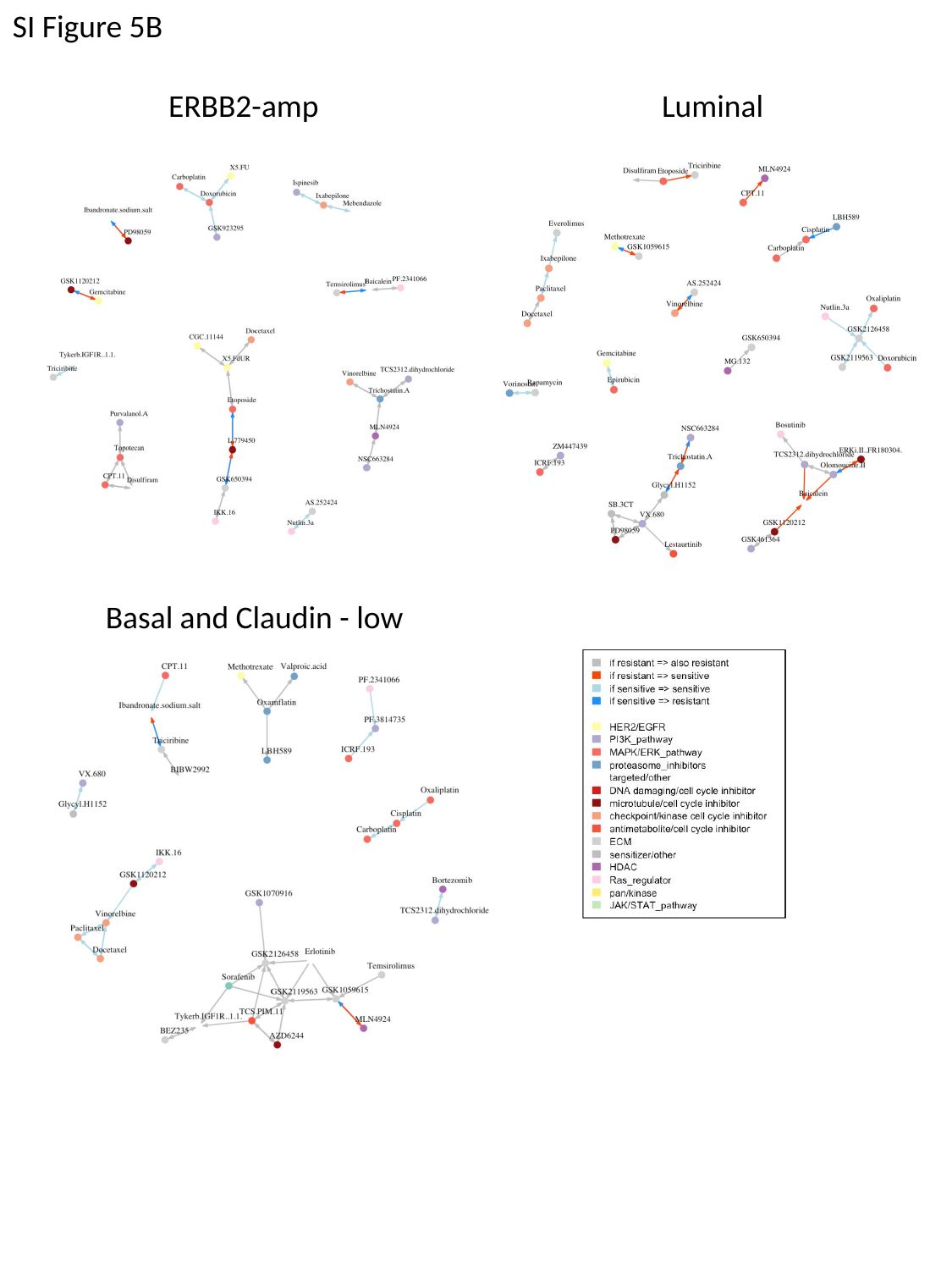

SI Figure 5B
ERBB2-amp
Luminal
Basal and Claudin - low

## Slide 11
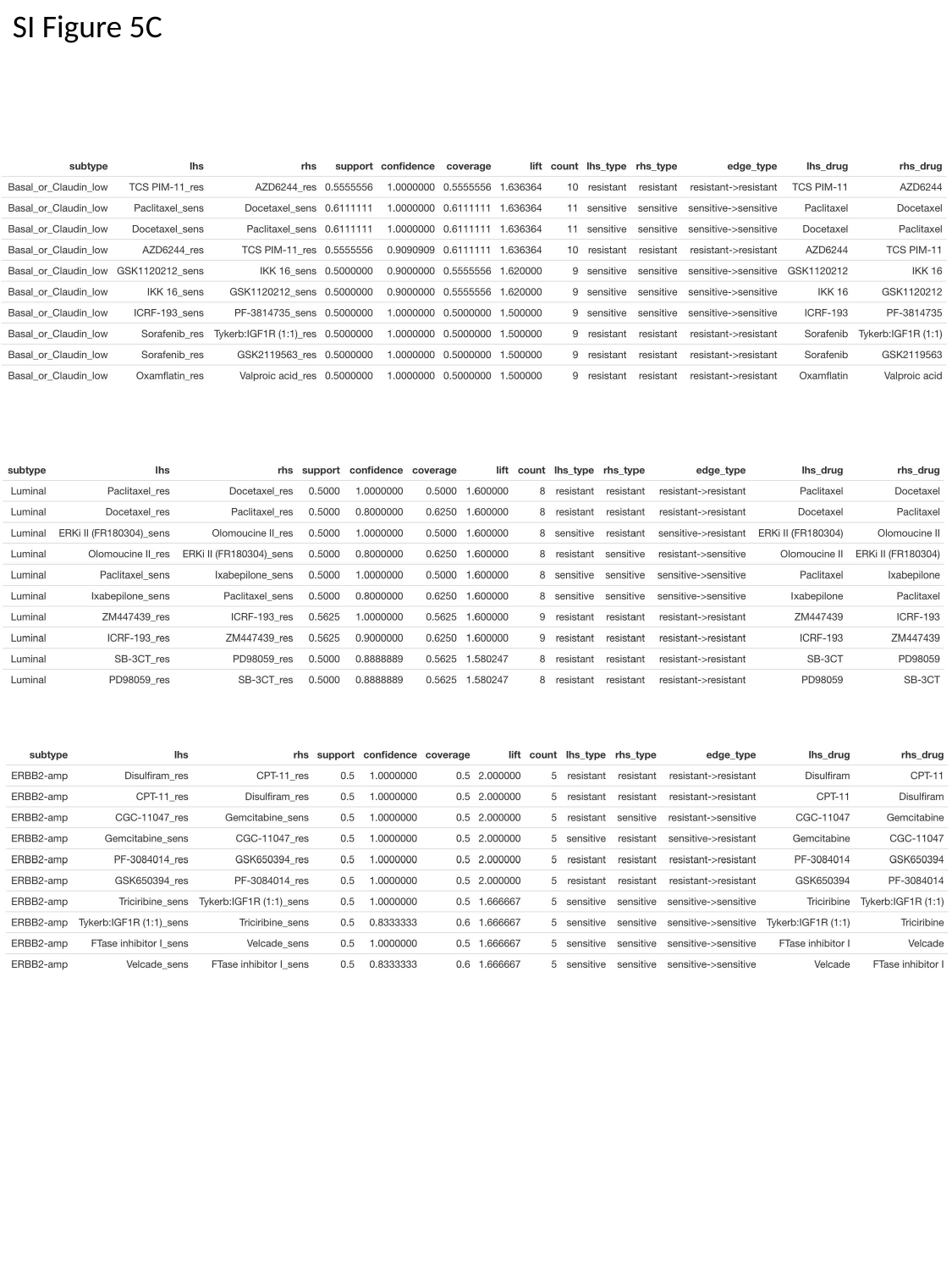

SI Figure 5C
